# Social Facilitation of Cognition in Rhesus Monkeys: Audience Vs. Coaction

**DOI:** 10.3389/fnbeh.2015.00328

**Published:** 2015-12-01

**Authors:** Amélie J. Reynaud, Carole Guedj, Fadila Hadj-Bouziane, Martine Meunier, Elisabetta Monfardini

**Affiliations:** ^1^ImpAct Team, Institut National de la Santé et de la Recherche Médicale, U1028, Centre National de la Recherche Scientifique, UMR5292, Lyon Neuroscience Research CenterLyon, France; ^2^Université de LyonLyon, France; ^3^Institut de Médecine EnvironnementaleParis, France

**Keywords:** social facilitation, social presence, audience, coaction, monkeys

## Abstract

Social psychology has long established that the mere presence of a conspecific, be it an active co-performer (coaction effect), or a passive spectator (audience effect) changes behavior in humans. Yet, the process mediating this fundamental social influence has so far eluded us. Brain research and its nonhuman primate animal model, the rhesus macaque, could shed new light on this long debated issue. For this approach to be fruitful, however, we need to improve our patchy knowledge about social presence influence in rhesus macaques. Here, seven adults (two dyads and one triad) performed a simple cognitive task consisting in touching images to obtain food treats, alone vs. in presence of a co-performer or a spectator. As in humans, audience sufficed to enhance performance to the same magnitude as coaction. Effect sizes were however four times larger than those typically reported in humans in similar tasks. Both findings are an encouragement to pursue brain and behavior research in the rhesus macaque to help solve the riddle of social facilitation mechanisms.

## Introduction

An individual's behavior changes merely because an observer is hovering nearby. Since Allport's 1924 *Social psychology* (Allport, 1924), this phenomenon is referred to as “social facilitation,” a historical misnomer as others' mere presence facilitates the emission of well-learned responses but impairs the acquisition of new responses (Zajonc, 1965). Social facilitation affects all human behaviors, from food consumption (Herman, 2015) to cognition (Bond and Titus, 1983). It was initially described in presence of co-performers (coaction effect) but subsequent research demonstrated that the presence of passive spectators (audience effect) suffices to produce the same change (Stroebe, 2012). Social psychology proposed several theoretical models to explain social facilitation (Strauss, 2002) but behavior alone cannot tease them apart. Recently, we showed that cognitive neuroscience and its nonhuman primate animal model, the rhesus macaque, could help solve this long debated issue by revealing which brain functions are altered by social presence (Monfardini et al., 2015). This novel approach is currently handicapped, however, by the fact that we do not know to which extent social facilitation in rhesus macaques parallels what social psychology has taught us about human social facilitation.

Social facilitation studies in rhesus macaques are few and far between; only three have been conducted over the last 80 years (Harlow and Yudin, 1933; Stamm, 1961; Ferrari et al., 2005). Two (Harlow and Yudin, 1933; Ferrari et al., 2005) show that macaques eat more in presence of co-eaters than alone (as do humans, Herman, 2015, or capuchin monkeys, Visalberghi and Addessi, 2000; Dindo and de Waal, 2007). The remaining one (Stamm, 1961) provides the proof of concept that the presence of a co-performer also helps macaques when food serves as a reward for a cognitive task rather than being simply made available for consumption (a change recently reported in capuchins as well; Dindo et al., 2009). The aim of the present study was to complete the patchy knowledge of social facilitation in macaques in two ways. First, we wanted to confirm Stamm's early (Stamm, 1961) demonstration of a social facilitation of cognition. Second, we wanted to establish that the present other needs not be a co-performer for the change to occur, and that a simple spectator suffices. To this aim, seven adult rhesus macaques (two dyads and one triad), trained to perform a simple cognitive task consisting in touching images to get food rewards, were alternatively tested alone vs. with a familiar companion serving as either a passive spectator or an active co-performer.

## Methods

### Subjects

Seven French-born rhesus macaques (*Macaca mulatta*), aged 4–11 years, participated in the study: two male-female dyads paired in adulthood, unsuccessfully in one case (dyad 1, the two animals were housed side by side but not together due to the male's unpredictable bouts of aggressiveness), successfully in the other case (dyad 2, the two animals had been living together for 3 years at the start of the experiment), and a triad of same-age females living together since birth. The monkeys had free access to water and received normal daily food rations of fresh fruits and monkey chow (40–130 kcal/kg/day) after testing completion. All animals were familiarized with handling and testing procedures for 4 weeks before the present data were collected. Data from the female triad were partially presented in Monfardini et al. (2015) (see next section for detail). Work complied with European Union Directive 2010/63/EU and was approved by French Animal Ethics Committee C2EA42-CELYNE.

### Procedure

**Figure 1:**
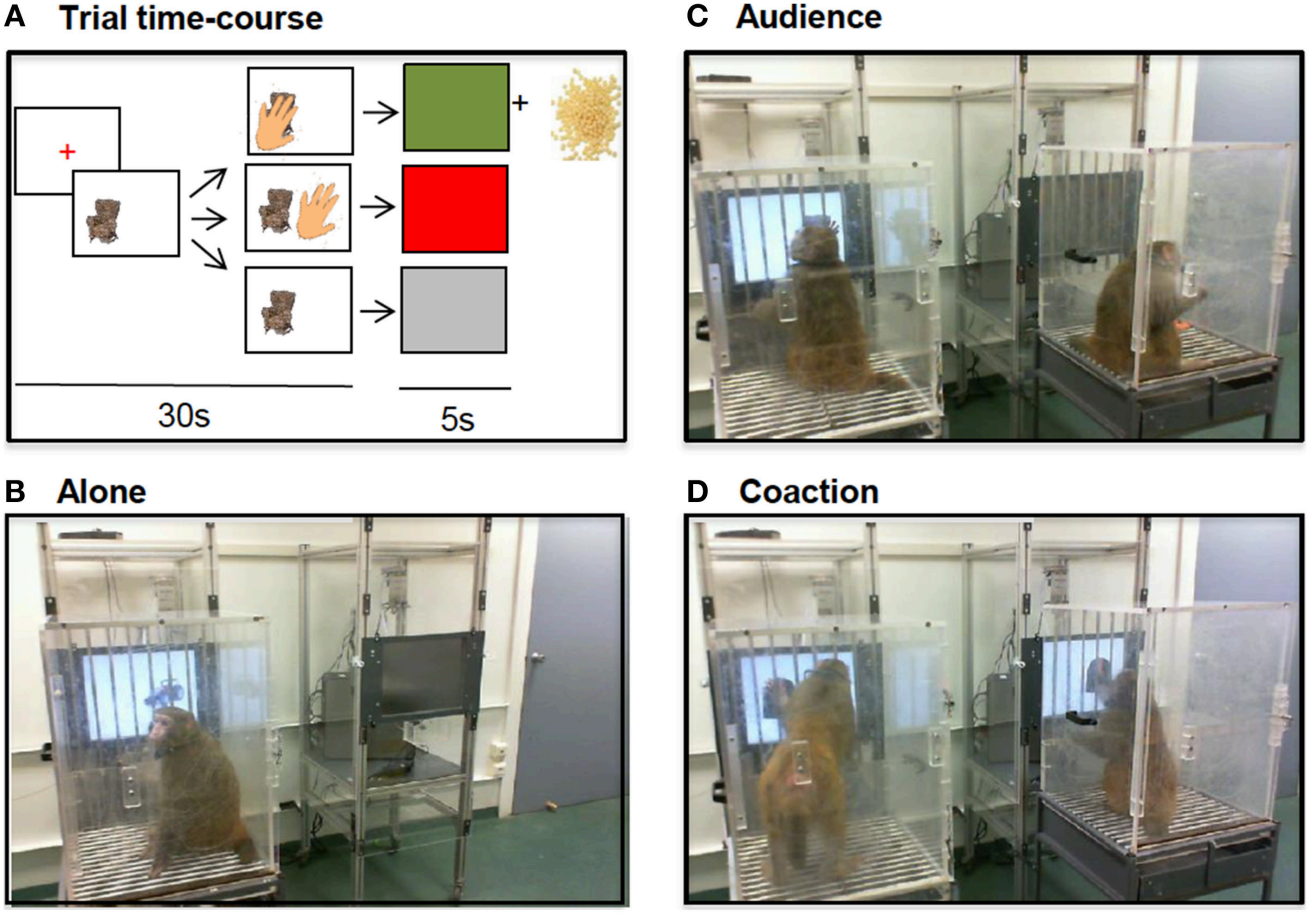
**Trial time-course (A)**. An image appeared on the screen. If the animal touched it within 30 s, a 5 s positive feedback appeared (green screen) and a reward (dry pasta beads) was delivered. Otherwise, a 5 s negative feedback appeared (a red screen for an incorrect touch, a gray screen for a no response) and no reward was delivered. Testing conditions **(B-D)**. The animals were tested alone **(B)**, in the presence of an idle companion **(C)** or in the presence of an active companion doing the same task **(D)**.

Seven pictures of neutral objects (e.g., an armchair, a farm tractor, a knit cap) were pseudo-randomly presented on the right or on the left side of the screen always in the same order. Touching the image led to a positive visual feedback plus a reward (~5 tiny beads of dry pasta); the trial time course (Figure 1A) allowed a maximum response rate of 8 responses/min. Each testing session lasted 15 min. For social testing, two animals were placed side by side; each monkey could see and hear the other one but not reach the other's screen or treats. The animals first alternated 1 day alone (Figure 1B), 1 day in the passive presence of a housemate (or neighbor for dyad 1; audience, Figure 1C), and then 1 day alone, 1 day in the presence of a housemate/neighbor doing the same task (coaction, Figure 1D). For the triad, half of the social sessions were carried out in the presence of one partner, the other half in the presence of the other partner; this group's audience scores were included in Monfardini et al. (2015) where they were correlated with changes in brain metabolism and changes in plasma cortisol level.

### Data analysis

Scores (response per min) in the alone condition were the same whether collected in alternation with the audience or with the coaction condition [paired *t*-test *t*_(6)_ = 1.2, *p* = 0.26]. They were therefore pooled into a single alone measure and compared to the two social conditions using a parametric ANOVA with the Huynh-Feldt correction for repeated measures followed by Bonferroni *post-hoc* tests for repeated measure. Effect size was estimated using Cohen's d (mean difference between social and alone scores divided by the pooled within-condition standard deviation), as well as Cohen's D_rm_ (d^*^SQRT(2^*^(1-r))), which corrects for the correlation r between measures in within-subject designs (Lakens, 2013).

Then, we calculated for each social session, the percent change in response rate relative to the animal's average performance during alone sessions (individual Δ scores). One sample *t*-tests were used to determine whether these individual Δ scores significantly differed from 0, and paired *t*-tests to compare audience and coaction effects for each individual. Finally, because the results revealed variable Δs across groups, Pearson's correlations were used to test the idea of a link between mean Δs over the two social conditions and the degree of intimacy between observer and observee as objectivized by the index: I = years of shared housing/years of age (Table 1).

**Table 1:** **Summary of the animals' characteristics, response rate, and Δ scores**.

**Group**	**Monkey**	**Years of age**	**Years of shared housing**	**Intimacy index I**	**Response rate (per min)**				**Mean Δ (% gain)**					
					**Alone**	**Audience**	**Coaction**	**Social**	**Audience**		**Coaction**		**Social**	
									**Δ**	***p***	**Δ**	***p***	**Δ**	***p***
Dyad 1	♂	9	0	0	1.9	2.6	3	2.8	37	0.003	57	0.000	46	0.000
	♀	11	0	0	2	3.2	3	3.1	60	0.000	52	0.004	57	0.000
Dyad 2	♂	8	3	0.4	0.5	0.9	1.7	1.2	76	0.040	237	0.019	144	0.002
	♀	11	3	0.3	0.4	1	1.5	1.2	144	0.010	283	0.030	200	0.001
Triad	♀1	4	4	1	1.7	3.9	3.7	3.8	191	0.000	175	0.000	123	0.000
	♀2	4	4	1	1.1	4.2	4.3	4.2	256	0.000	269	0.000	282	0.000
	♀3	4	4	1	0.7	3.1	2	2.6	524	0.000	298	0.001	305	0.000
Average		7	3	0.5	1.2	2.7	2.8	2.7	184		196		165	
sem		1	1	0.2	0.3	0.5	0.4	0.4	64		40		39	

*Social, scores for audience and coaction taken together; Mean Δ, percent change in response rate relative to the animal's average performance during alone sessions; p-values, significant facilitation revealed by one-sample t-tests relative to zero. Within each group, the animals are listed in hierarchical order, the top-ranking animal appearing first. To the best of our knowledge, there was no genetic link between the members of each group*.

## Results

**Figure 2:**
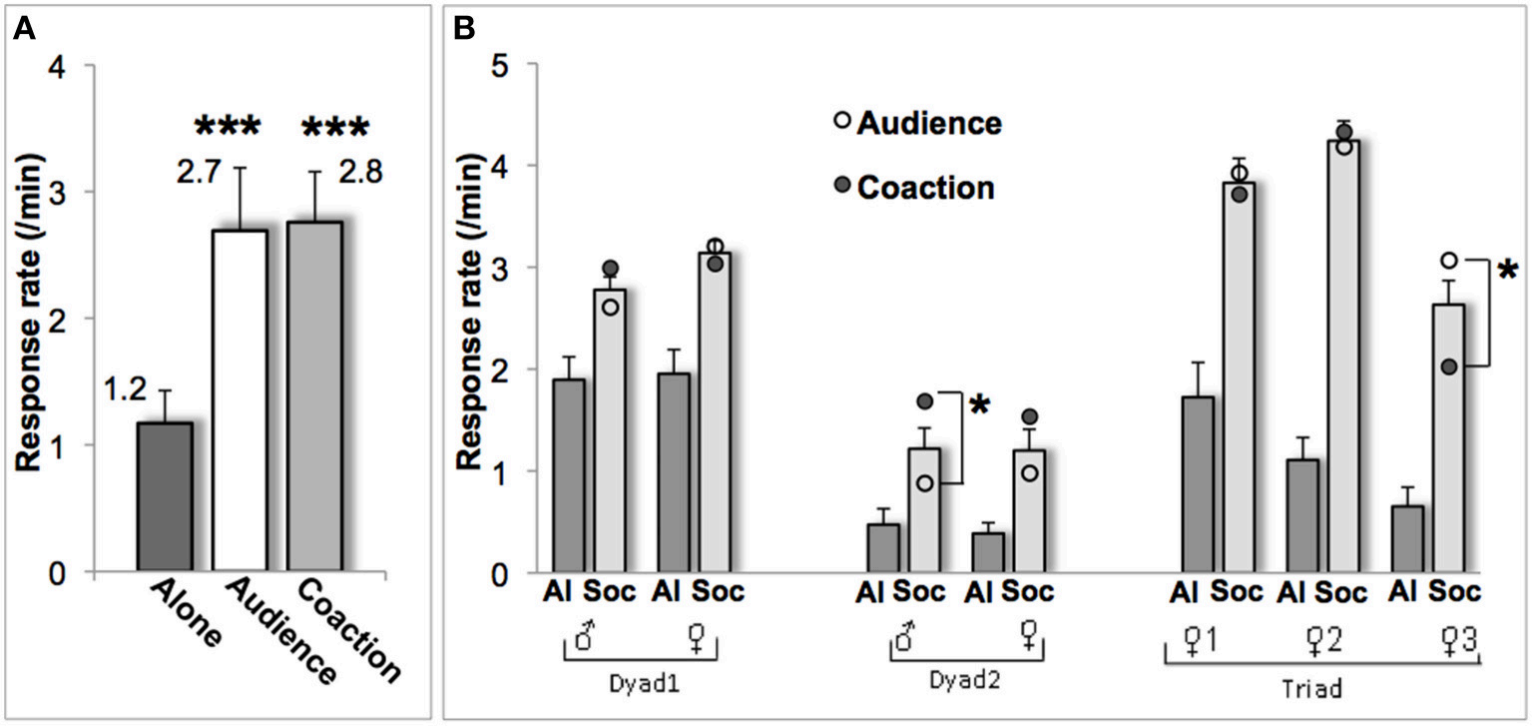
**Response rate (mean ± sem) for the group (A) and for each individual (B)**. In **(B)**, scores are illustrated for the alone condition (*Al*, dark gray bars), the two social conditions taken together (*Soc*, light gray bars), and for audience (o) and coaction (•) separately. ^***^p ≤ 0.001, ^*^p ≤ 0.05.

The animals completed on average 19 sessions (range 16–20) in the alone condition, 15 sessions (10–21) in the audience condition, and 11 sessions (8–17) in the coaction condition. The monkeys touched the images on the screen to obtain a food treat twice more often under audience and coaction than under alone testing [*F*_(2, 12)_ = 16.0; *p* = 0.001; Figure 2A]. Each social condition differed from the alone condition (audience: *p* = 0.009, coaction: *p* = 0.002), while not differing from each other (*p* = 0.80). Effect sizes were systematically superior to the 0.8 value reflecting a large effect (Lakens, 2013). Cohen's d amounted to 1.4 for audience and 1.8 for coaction, and Cohen's D_rm_ to 1.3 for audience and 1.6 for coaction.

At the individual level, each of the seven monkeys displayed a change (mean Δ) significantly differing from zero in both the audience and coaction conditions (all *p*'s ≤ 0.04). In most cases (5/7), audience and coaction effects were indistinguishable. A significant difference emerged in two monkeys though (Figure 2B): the (assertive) male of dyad 2 performed better in the coaction condition [*t*_(17)_ = 2.1, *p* = 0.05], whereas the (subdued) bottom-ranking female of the triad performed better in the audience condition [*t*_(34)_ = 2.3, *p* = 0.03]. Mean Δs over the two social conditions were positively correlated with intimacy scores (*r* = 0.75, *p* = 0.05). The largest facilitation occurred in the triad of lifetime companions, the smallest in the dyad of neighbors, while the dyad of adulthood companions fell in between (Table 1).

## Discussion

The present study confirms an early demonstration of social facilitation of cognition in rhesus macaques (Stamm, 1961), and demonstrates that, as in humans, coaction is not necessary for this phenomenon to occur. The mere presence of a passive spectator produces the very same change. Effect sizes were four times larger than those reported for equivalent tasks in humans (Bond and Titus, 1983). Some variations across individuals and groups hinted at potential moderators of social facilitation for future studies to investigate.

The present coaction effect adds to the rare earlier evidence that macaques (and capuchins) are socially facilitated also when food serves as a reward for a task, rather than being simply made available for consumption (Stamm, 1961; Dindo et al., 2009). The present audience effect provides a convincing example of the mere presence effect in nonhuman animals. In the 1960s, social psychologist Zajonc gave equal weight to human data and nonhuman data from a variety of animals including cockroaches, chickens, or rats, considering that “*a social psychology confined to man is as parochial as a chemistry confined to gold*” (Rajecki, 2010). Later, however, the capacity of animal studies to arrange (i) a presence condition without social learning of some sort and (ii) a solitary condition that is not a source of stress was called into question (Guerin and Innes, 2009). Here, the “present other” was of no help to solve the task at hand, so social learning (whether cueing, contagion, or imitation) cannot explain the social facilitation effect. A stress-related explanation can also reasonably be ruled out as we compared the cortisol response elicited in the present female triad by the alone *vs*. the audience condition, and found no difference (Monfardini et al., 2015).

The present social facilitation is in line with the enhanced response rate described in simple cognitive tasks in humans (Bond and Titus, 1983). Likewise, the fact that both audience and coaction facilitate performance is consistent with human studies reporting equipotentiality of the two effects in the cognitive domain (Fonseca and Garcia-Marques, 2013). These similarities suggest that social facilitation is a form of social influence that arose through evolution and operates regardless of the level of language, culture, or intelligence. This strengthens the idea that rhesus macaques constitute a valuable animal model of human social facilitation and that brain and behavior studies in this classical neuroscience model (Capitanio and Emborg, 2008) could provide the mean for a fresh look at one of the oldest topics in social psychology (Stroebe, 2012).

As evoked in the Introduction, neuroimaging could help solve the riddle of social facilitation mediator (Monfardini et al., 2015). Monkeys present an advantage over humans in this new endeavor: the large effect sizes they display (> 1.2 standard deviation unit), well above the modest effect sizes typically observed in humans (~0.3 standard deviation unit; Bond and Titus, 1983). The amplitude of the neural changes revealed by neuroimaging being correlated with the amplitude of the accompanying behavioral changes (Monfardini et al., 2015), the neural signature of social facilitation could be easier to uncover in monkeys than in humans. Regarding behavior, rhesus macaques might help explore moderators of social facilitation that have heretofore been neglected. The variations observed here, across groups (greater facilitation in lifetime female companions), and across individuals (greater audience effect in a subdued female, vs. greater coaction effect in an assertive male), respectively suggest a moderating influence of the observer-observee degree of familiarity (Herman, 2015), and a moderating influence of gender and/or personality traits (Uziel, 2007) for future studies to investigate in larger samples of animals.

## Funding

INSERM, CNRS, UCBL, Institut de Médecine Environnementale, NEURODIS Foundation, ANR-O8-BLAN-0068-1, James S. McDonnell Foundation, LABEX CORTEX-ANR-11-LABX-0042.

### Conflict of interest statement

The authors declare that the research was conducted in the absence of any commercial or financial relationships that could be construed as a potential conflict of interest.
